# Reduced resting-state brain functional network connectivity and poor regional homogeneity in patients with CADASIL

**DOI:** 10.1186/s10194-019-1052-6

**Published:** 2019-11-11

**Authors:** Jingjing Su, Shiyu Ban, Mengxing Wang, Fengchun Hua, Liang Wang, Xin Cheng, Yuping Tang, Houguang Zhou, Yu Zhai, Xiaoxia Du, Jianren Liu

**Affiliations:** 1 Department of Neurology, Shanghai Ninth People’s Hospital, Shanghai Jiao Tong University School of Medicine, 639 Zhizaoju Road, Shanghai, 200011 People’s Republic of China; 2 Shanghai Key Laboratory of Magnetic Resonance and Department of Physics, School of Physics and Materials Science, East China Normal University, 3663 North Zhongshan Road, Shanghai, 200062 People’s Republic of China; 3 College of Medical Imaging, Shanghai University of Medicine & Health Sciences, 279 Zhouzhu Highway, Shanghai, 201318 People’s Republic of China; 4 PET Center, Huashan Hospital, Fudan University, 518 East Wuzhong Road, Shanghai, 200235 People’s Republic of China; 5 Department of Neurology, Huashan Hospital, Fudan University, 12 Middle Wulumuqi Road, Shanghai, 200040 People’s Republic of China; 6 Department of Geriatrics Neurology, Huashan Hospital, Fudan University, 12 Middle Wulumuqi Road, Shanghai, 200040 People’s Republic of China

**Keywords:** Functional network connectivity, Regional homogeneity, CADASIL, Resting-state fMRI, Visuomotor behaviors

## Abstract

**Background:**

Cerebral autosomal dominant arteriopathy with subcortical infarcts and leukoencephalopathy (CADASIL) manifests principally as a suite of cognitive impairments, particularly in the executive domain. Executive functioning requires the dynamic coordination of neural activity over large-scale networks. It remains unclear whether changes in resting-state brain functional network connectivity and regional homogeneities (ReHos) underly the mechanisms of executive dysfunction evident in CADASIL patients.

**Methods:**

In this study, 22 CADASIL patients and 44 matched healthy controls underwent resting-state functional magnetic resonance imaging (fMRI). Independent component analysis (ICA) was used to measure functional brain network connectivity, and ReHos were calculated to evaluate local brain activities. We used seed-based functional connectivity (FC) analyses to determine whether dysfunctional areas (as defined by ReHos) exhibited abnormal FC with other brain areas. Relationships among the mean intra-network connectivity z-scores of dysfunctional areas within functional networks, and cognitive scores were evaluated using Pearson correlation analyses.

**Results:**

Compared to the controls, CADASIL patients exhibited decreased intra-network connectivity within the bilateral lingual gyrus (LG) and the right cuneus (CU) (thus within the visual network [VIN)], and within the right precuneus (Pcu), inferior frontal gyrus (IFG), and precentral gyrus (thus within the frontal network [FRN]). Compared to the controls, patients also exhibited significantly lower ReHos in the right precuneus and cuneus (Pcu/CU), visual association cortex, calcarine gyri, posterior cingulate, limbic lobe, and weaker FC between the right Pcu/CU and the bilateral parahippocampal gyrus (PHG), and between the right Pcu/CU and the right postcentral gyrus. Notably, the mean connectivity z-scores of the bilateral LG and the right CU within the VIN were positively associated with compromised attention, calculation and delayed recall as revealed by tests of the various cognitive domains explored by the Mini-Mental State Examination.

**Conclusions:**

The decreases in intra-network connectivity within the VIN and FRN and reduced local brain activity in the posterior parietal area suggest that patients with CADASIL may exhibit dysfunctional visuomotor behaviors (a hallmark of executive function), and that all visual information processing, visuomotor planning, and movement execution may be affected.

## Introduction

Cerebral autosomal dominant arteriopathy with subcortical infarcts and leukoencephalopathy (CADASIL) is the most common form of hereditary, subcortical vascular dementia; the condition is attributable to pathogenic mutations in the NOTCH3 gene of chromosome 19. Usually, the earliest signs are recurrent strokes and cognitive deficits dominated by early impairment of executive functions, and it is commonly associated with deficits in attention and memory [1, 2]. Such dysfunction was present in all subjects aged 35–73 years in a study that recruited 42 consecutive, symptomatic CADASIL patients [2]. The cognitive impairments including executive abnormalities in CADASIL are mainly characterised by frontal-like symptoms such as poor attention, perseverations and apathy, and memory impairment associated with pyramidal signs, gait difficulties, pseudobulbar palsy, and sphincter incontinence [3]. Cognitive decline increases with age, and is associated with progressive deterioration of instrumental activities, visuospatial abilities, visual memory, and reasoning [2]. After the age of 60 years, significant deficits in all cognitive domains are evident, severely compromising independent daily living and even causing death [2]. No effective treatment is known [1]. Thus, an understanding of the mechanisms underlying executive dysfunction in patients with this devastating disorder will increase our understanding of this condition and pave the way for new therapeutic trials.

Age, male sex, active smoking, and systolic blood pressure are strong independent risk factors for clinical deterioration (including executive deficits) in patients with CADASIL [4–7]. Of these, smoking and systolic blood pressure are modifiable [6, 7]. Quantitative magnetic resonance imaging (MRI) has revealed close associations between measured parameters and the executive functions of patients with CADASIL. Both the overall lacunar lesional burden and extent of brain atrophy, as reflected by the normalized brain volume yielded by structural image evaluation using normalization of atrophy software, significantly influence the severity of executive performance deterioration and cognitive decline as assessed by executive scores [4, 8–10]. The global cortical atrophy scale (another measure of brain volume change) was developed by Pasquier to rate the extent of atrophy in 13 brain MRI regions on a visual scale, and predict poor executive performance [11]. The corpus callosum area as determined by three-dimensional (3D)-T1 MRI sequences (using well-validated methodology) is independently correlated with reaction time during the performance of a simple task, and can serve as a proxy of early cognitive and behavioral changes in patients with CADASIL [12, 13]. Diffusion tensor imaging (DTI) yields useful information on the extent of white matter (WM) tract damage; the cingulum bundle seems to be important in terms of maintaining the anteroposterior connections necessary for executive functioning in nondemented CADASIL patients [14]. Furthermore, DTI abnormalities in white and deep gray matter that appear normal using other imaging modalities have been detected in nondemented CADASIL patients; these abnormalities correlated particularly strongly with executive function [15]. Thus, it may be that widespread WM damage explains the executive dysfunction apparent during the early stage of CADASIL; this suggestion is (at least in part) consistent with pathological data derived from post-mortem brains. Extensive pathological WM lesions were evident in all brain regions, particularly the frontal motor cortex, suggesting the disruption of either the cortico-cortical or cortico-subcortical networks of frontal lobe WM, which may explain the observed motor deficits and executive dysfunctions [16].

Brain functional MRI (fMRI) has shed light on the causal mechanisms of executive dyscognition in CADASIL patients. A study evaluating the association between resting-state functional network connectivity and cognitive performance indicated that the status of two frontoparietal components correlated with executive performance [17]. Our recent resting-state amplitude of low-frequency fluctuation (ALFF) analyses revealed negative associations between the ALFF values of the left cerebellar anterior and posterior lobes, and executive function scores [18]. An event-related Go/No-go task study on CADASIL patients revealed lower blood oxygen level-dependent (BOLD) effects in the alerting network and in areas involved in executive functions, possibly reflecting hemodynamic responses that developed secondary to small-vessel changes [19].

Thus, although the scores on various indices appear to correlate with the extent of executive disability in CADASIL patients, it is currently considered that human executive functions are mediated by the dynamic interplay of large-scale brain networks [20]. However, it remains largely unknown whether changes in resting-state brain functional network connectivity and regional homogeneities (ReHos) can provide novel insights into the executive dysfunction mechanisms in play in CADASIL patients. Resting-state functional network connectivity evaluated via independent component analysis (ICA) has been used to define several, intrinsic functional networks [17]. In addition, ReHos can be used to identify local features of spontaneous brain activity when the Kendall coefficient of concordance (KCC) is employed during synchrony evaluation of BOLD time series. The intra-network connectivity within brain networks and ReHos have been shown to correlate with behavioral measures in both healthy subjects and those with neuropsychiatric conditions [21]. Based on the above mentioned abnormalities in executive behaviors and brain structure [6–15], we hypothesized that both brain functional network connectivity and ReHos would be altered in CADASIL patients. To this end, we used resting-state fMRI to explore changes in brain functional networks as assessed by ICA and local ReHos.

## Methods

### Participants

We recruited 22 patients with CADASIL (13 males and 9 females) from 11 families; all patients were referred to the Department of Neurology at Shanghai Ninth People’s Hospital, Shanghai Jiao Tong University School of Medicine between May 2015 and August 2017. The probands for each family were selected based on the presence of recurrent stroke, vascular dementia, and leukoencephalopathy; all were eventually genetically diagnosed with CADASIL. Subsequently, other family members underwent genetic screening, and those with genetic diagnoses of CADASIL were also included. Of the 22 cases, 4 were asymptomatic whereas the remaining 18 suffered from stroke, headache, and cognitive impairments. The exclusion criteria were any other neurodegenerative disorder or severe untreated depression or anxiety. Forty-four healthy matched controls (26 males and 18 females) served as the control group. None had a history of stroke, headache, or cognitive impairment; their family members lacked any history of cerebrovascular disease and did not exhibit vascular disease risk factors. All neurological and psychiatric diseases were excluded based on clinical examination and MRI evaluation; lacunae and WM lesions were absent.

### Clinical assessment

We recorded sex; age; family medical history; any history of stroke, transient ischemic attack (TIA), or headache; and vascular disease risk factors (e.g., hypertension, diabetes, coronary heart disease, hyperlipidemia, and smoking). Cognitive scores on the Mini-Mental State Examination (MMSE) and Montreal Cognitive Assessment (MoCA) were noted. MMSE and MoCA allowed the assessments of different cognitive domains. The three-word delayed recall test from MMSE was used to measure the degree of cognitive impairment and assess the domain of episodic memory. In the delayed recall task, the research staff read out loud 3 unrelated words to the subjects and the subjects were informed to be asked to recall the words later. Its scores ranged from 0 to 3, reflecting the number of words correctly recalled. A score ≤ 1 identified the participants with poor delayed recall [22, 23]. Furthermore, a neurologist evaluated the neurological deficits of patients using the National Institute of Health Stroke Scale (NIHSS) and the modified Rankin scale (mRs). State depression and anxiety were assessed using the Hamilton Depression Scale (HAMD) and the Hamilton Anxiety Scale (HAMA), respectively.

### MRI

MRI scans were performed at the East China Normal University using a 3.0-T Siemens Trio Tim system fitted with a 12-channel head coil. Head movements were minimized using custom-fitted foam pads. The structural MRI scan included T1- and T2-weighted and FLAIR imaging. The FLAIR sequence parameters were repetition time of 9000 ms; echo time of 93 ms; field-of-view of 199 × 220 mm^2^; 30 slices; and slice thickness of 3.5 mm. The parameters of the T2-weighted imaging were as follows: turber spin echo dark fluid sequence, repetition time of 5500 ms; echo time of 83 ms; field-of-view of 220 × 220 mm^2^; 35 slices; and slice thickness of 3 mm. The resting-state fMRI images were acquired using a T2*-weighted gradient-echo, echo-planar, and imaging pulse sequence with the following parameters: repetition time of 2000 ms; echo time of 30 ms; flip angle of 90°; field-of-view of 220 × 220 mm^2^; matrix size of 64 × 64; 33 slices; slice thickness of 3.5 mm; and 210 volumes. Whole-brain anatomical volume was obtained using a high-resolution, T1-weighted, 3D, magnetization-prepared, rapid-acquisition, gradient-echo pulse sequence with the following parameters: repetition time of 2530 ms; echo time of 2.34 ms; field-of-view of 256 × 256 mm^2^; 192 slices; slice thickness of 1 mm; and flip angle of 7°.

### Assessments of lacunae and WM lesions

The presence of lacunae was assessed using 3D, T1 images. Hypointense lesions on T1-weighted imaging with a signal identical to cerebrospinal fluid, sharp delineation, and diameter > 2 mm were selected as previously reported [24]. The number of lacunae was thereafter counted. WM lesions were evaluated using FLAIR sequences and defined as WM areas with increased signal intensity on FLAIR sequences. The severity of WM hyperintensity was assessed visually on axial FLAIR images according to the modified Fazekas scale [25], the most widely used scale to describe WM hyperintensity severity. This scale divided WM hyperintensities into periventricular and deep categories. Periventricular WM hyperintensities were graded according to the following patterns: 0 = absent, 1 = caps or a pencil-thin lining, 2 = a smooth halo, and 3 = irregular WM hyperintensities extending into the deep WM. Deep WM hyperintensities were graded according to the following patterns: 0 = absent or single punctate foci, 1 = multiple punctate foci, 2 = beginning confluence of foci, and 3 = large fused foci. The total scores were acquired by adding the periventricular and deep WM hyperintensity scores.

### Resting-state fMRI data preprocessing

The resting-state fMRI data were preprocessed using the Data Processing and Analyses of Brain Imaging software (a newly developed toolbox), and loaded into statistical, parametric mapping software ver. 12 (http://www.fil.ion.ucl.ac.uk/spm/software/spm12) [26]. Thus, manual manipulations prior to data analysis were minimized. The preprocessing steps featured slice-timing correction, realignment of functional data to the first images, and co-registration of functional and structural images. All data were spatially normalized to the standard Montreal Neurological Institute space. Functional images were subjected to Gaussian spatial smoothing (6 mm full-width at half-maximum). Subjects exhibiting head movements > 2 mm were excluded from the analyses, and signals from cerebral WM and cerebrospinal fluid were removed using a general linear model.

### Data analyses

#### ICA and resting-state networks analyses

We used ICA to preprocess data using the Group ICA of fMRI toolbox (GIFT 4.0a, http://icatb.sourceforge.net/), which runs an Infomax algorithm. The preprocessed group data were decomposed into 31 spatial independent components (ICs). The data were concatenated and reduced using two-stage principal component analysis (PCA) and ICs, and then calculated using the Infomax algorithm. The GICA-3 back-reconstruction step was used to separate single-subject components from the set of aggregate components calculated in the previous step. Finally, for all subjects, the acquired spatial component maps were converted into z-score maps.

The correlation analyses were performed between the 31 spatial ICs and 7 resting-state networks using Pearson correlation as previous study had demonstrated [27]. The brain networks were divided based on their anatomical and functional properties and included basal ganglia, auditory, sensorimotor, visual, default-mode, attentional, and frontal networks. We then obtained a matrix of Pearson correlation coefficients and applied a threshold to the correlation coefficients at *r* > 0.4, indicating that the ICs belonged to the corresponding brain networks.

#### ReHo analyses

ReHo analyses were based on the preprocessed data described above. Individual ReHo maps were generated by calculating the KCC concordance of each time series to those of its 26 nearest neighbors for each voxel [21]. To eliminate any effect of individual differences, the ReHo of each voxel was converted into a z-score by subtracting the average ReHo value and dividing the value thus obtained by the standard deviation of the whole-brain ReHo map, yielding a standard ReHo value.

#### Seed-based functional connectivity (FC) analyses

We used seed-based FC analyses to explore whether dysfunctional areas (as revealed by ReHos) exhibited abnormal FC with other brain areas. The former (dysfunctional) brain areas served as the seed regions. The mean time series of each seed region was correlated with the time series of each whole-brain voxel, yielding FC maps that were converted into z-score maps using the Fisher Z-transformation.

#### Correlational analyses

Individual mean ICA z-scores and ReHo z-scores for the surviving CADASIL clusters were extracted. Associations between the mean z-scores for ICA and ReHo in the significantly altered brain regions and the clinical measures, including the MMSE, MoCA, NIHSS, mRs, HAMD, and HAMA, were assessed. In order to explore different cognitive domains in the MMSE and MoCA, we used subanalyses to detect specific correlations with mean ICA z-scores, and ReHo z-scores of the surviving CADASIL clusters.

#### Statistical analyses

Maps showing significant differences (i.e., ICA, ReHos, and seed-based FC maps of the 22 CADASIL patients and 44 controls) were compared using voxel-wise two-sample *t*-tests, with age and sex as covariates, after applying a brain mask. To deal with the issue of multiple comparisons, the aforementioned statistical maps were assigned thresholds at *p* < 0.001 (thus at the voxel level), and family wise errors (FWE) were corrected to a *p*-value < 0.05 at the cluster level. Then the surviving clusters were analyzed. Pearson correlation analyses were used to identify correlations with clinical scores. The clinical data of the two groups were compared using Pearson’s chi-square test to explore possible sex-based differences, and the independent-samples *t*-test was used to evaluate age as well as the MMSE, MoCA, HAMD, and HAMA scores. *P* < 0.05 was considered statistically significant.

## Results

### Clinical data

The demographic and clinical data of the CADASIL patients and control groups were listed in Table 1. Sex, age at the time of the visit, and the median depression and anxiety symptom scores did not differ between the groups. However, there were differences in the cognitive scores on the MMSE and MoCA. The different subscores according to the different cognitive domains of MMSE and MoCA in CADASIL patients had also been showed in Tables 2 and 3.

**Table 1:** Demographic and clinical characteristics of CADASIL patients and controls

	CADASIL (*n* = 22)	Controls (*n* = 44)	*P* values
Male/Female	13/9	26/18	1
Age at visit (years), mean ± SD	48.9 ± 14.2	48.4 ± 13.7	0.890
Age at first symptom (years), mean ± SD	44.6 ± 12.4	–	–
Family history, n (%)	20 (90.9)	–	–
Previous stroke or TIA (times), mean ± SD	2.0 ± 2.3	–	–
Headache, n (%)	4 (18.2)	–	–
Vascular disease risk factors, n (%)	11 (50.0)	–	–
MMSE score, mean ± SD	23.5 ± 5.7	28.2 ± 1.3	0.001
MoCA score, mean ± SD	20.4 ± 7.6	27.8 ± 1.3	0.000
NIHSS score, mean ± SD	1.4 ± 2.1	–	–
mRs score, mean ± SD	1.5 ± 1.2	–	–
HAMD score, mean ± SD	6.8 ± 6.4	3.9 ± 1.7	0.052
HAMA score, mean ± SD	5.7 ± 5.4	3.8 ± 1.7	0.112

Values are means ± SDs or numbers with percentages

*CADASIL* Cerebral autosomal dominant arteriopathy with subcortical infarcts and leukoencephalopathy, *SD* Standard deviation, *TIA* Transient ischemic attack, *MMSE* Mini-mental state examination, *MoCA* Montreal cognitive assessment, *NIHSS* National Institute of Health Stroke Scale, *mRs* Modified rankin scale, *HAMD* Hamilton depression scale, *HAMA* Hamilton anxiety scale

**Table 2:** The different subscores of MoCA in CADASIL patients

Patients	MoCA						
	Visuospatial/execution	Naming	Attention	Language	Abstraction	Delayed recall	Orientation
1	4	3	6	2	2	5	6
2	5	3	6	3	2	5	6
3	5	3	6	3	2	5	6
4	5	3	6	2	2	5	6
5	4	3	6	3	2	5	6
6	4	3	6	3	2	2	6
7	3	3	4	2	2	3	6
8	1	2	1	0	1	0	3
9	4	3	4	2	2	0	6
10	3	2	4	1	2	3	6
11	4	3	6	3	2	3	6
12	4	1	4	1	2	0	6
13	2	1	1	0	1	0	1
14	0	1	3	1	1	1	2
15	2	3	6	1	2	2	6
16	0	2	3	0	2	1	3
17	4	3	6	1	2	2	6
18	0	2	1	0	0	0	5
19	2	2	5	1	1	1	4
20	3	3	5	1	1	0	5
21	3	3	6	1	1	4	6
22	1	3	3	1	2	0	2

*MoCA* Montreal cognitive assessment, *CADASIL* Cerebral autosomal dominant arteriopathy with subcortical infarcts and leukoencephalopathy

**Table 3:** The different subscores of MMSE in CADASIL patients

Patients	MMSE										
	Time orientation	Location orientation	Registration	Attention/ calculation	Delayed recall	Naming	Retelling	Auditory comprehension	Reading comprehension	Expression	Visuospatial ability
1	5	5	3	5	3	2	1	3	1	1	1
2	5	5	3	5	3	2	1	3	1	1	1
3	5	5	3	5	3	2	1	3	1	1	1
4	5	5	3	5	3	2	1	3	1	1	1
5	5	5	3	4	3	2	1	3	1	1	1
6	5	5	3	5	3	2	1	3	1	1	1
7	5	5	3	1	3	2	0	3	1	0	1
8	3	2	3	0	1	2	1	3	1	0	0
9	5	3	3	1	2	2	1	3	1	1	1
10	5	5	3	4	3	2	1	3	1	0	0
11	5	5	3	5	2	2	1	3	1	1	1
12	5	5	3	1	2	2	1	0	1	1	0
13	1	0	2	0	1	2	0	2	1	0	0
14	1	5	3	1	1	1	1	2	1	0	0
15	5	5	3	4	1	2	1	1	1	0	1
16	3	2	2	1	1	2	1	3	1	0	0
17	5	5	3	5	1	2	1	3	1	1	1
18	4	5	3	0	0	2	0	2	1	0	0
19	4	2	3	2	1	2	1	3	1	0	0
20	4	2	3	2	1	2	1	3	1	0	0
21	5	5	3	4	3	2	1	3	1	1	1
22	1	3	3	1	3	2	1	0	1	1	1

*MMSE* Mini-mental state examination, *CADASIL* Cerebral autosomal dominant arteriopathy with subcortical infarcts and leukoencephalopathy

### ICA

**Fig. 1:**
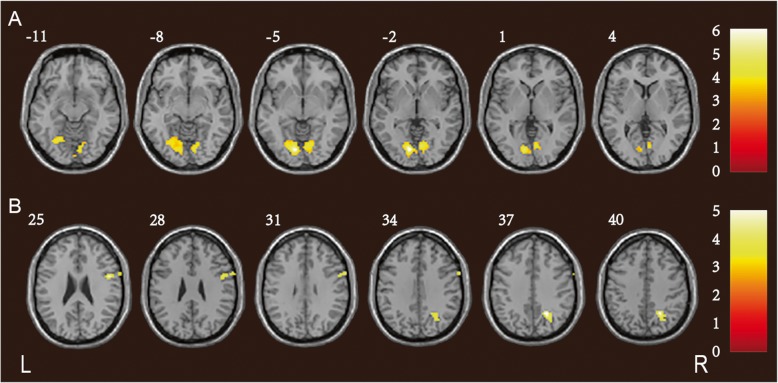
Reduced intra-network connectivity in patients with CADASIL. The CADASIL group exhibited decreased activity of the bilateral LG and the right CU within the VIN (**a**); and of the right Pcu, IFG, and precentral gyrus within the FRN (**b**), compared to the control group. The *t-*values are color-coded. CADASIL: cerebral autosomal dominant arteriopathy with subcortical infarcts and leukoencephalopathy; LG: lingual gyrus; CU: cuneus; VIN: visual network; Pcu: precuneus; IFG: inferior frontal gyrus; FRN: frontal network

Compared to the controls, patients with CADASIL exhibited decreased intra-network connectivity in the bilateral lingual gyrus (LG) and the right cuneus (CU) of the visual network (VIN), and within the right precuneus (Pcu), inferior frontal gyrus (IFG), and precentral gyrus of the frontal network (FRN) (Fig. 1; Table 4).

**Table 4:** Significant inter-group ICA differences between CADASIL patients and controls

Predominant cluster regions	Cluster size	Peak *T* value	MNI coordinates			Cluster level *P*FWE-corr
			x	y	z	
CADASIL < controls in VIN						
Bilateral LG	348	−6.19	−9	−78	−3	0.000
Right CU						
CADASIL < controls in FRN						
Right Pcu	59	−5.18	24	−57	36	0.002
Right IFG	67	−5.05	48	3	21	0.001
Right precentral gyrus						

The surviving ICA clusters were assigned thresholds of *p* < 0.001 and FWE-corrected to *p* < 0.05 at the cluster level

*ICA* Independent component analysis, *CADASIL* Cerebral autosomal dominant arteriopathy with subcortical infarcts and leukoencephalopathy, *MNI* Montreal Neurological Institute, *FWE* Family wise errors, *VIN* Visual network, *LG* Lingual gyrus, *CU* Cuneus, *FRN* Frontal network, *Pcu* Precuneus, *IFG* Inferior frontal gyrus

### ReHos and seed-based FC

**Fig. 2:**
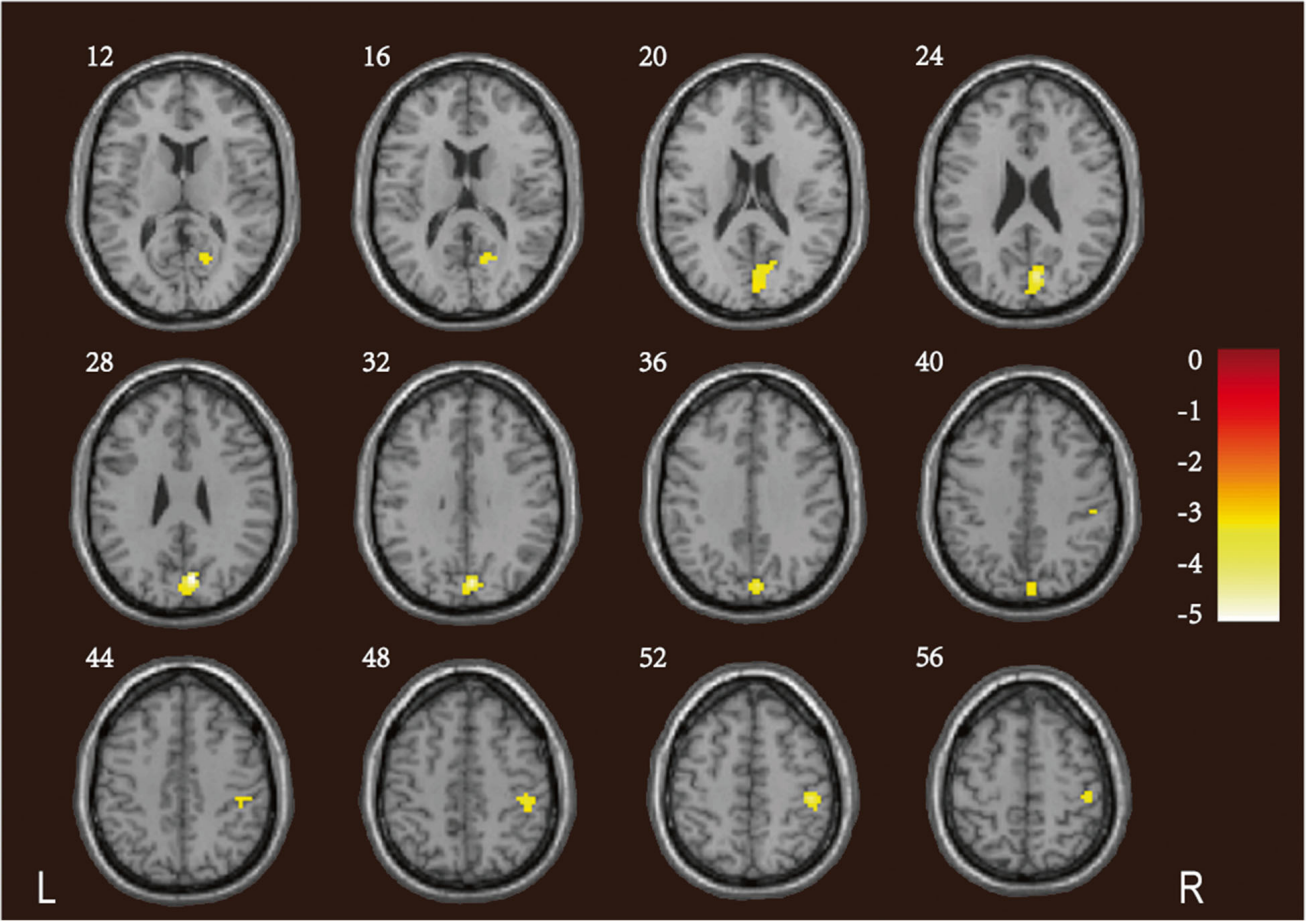
Reductions in the ReHos in patients with CADASIL. The CADASIL group exhibited significantly lower ReHos in the right Pcu/CU, visual association cortex, calcarine gyri, posterior cingulate and limbic lobe, compared to controls. The *t-*values are color-coded. ReHo: regional homogeneity; CADASIL: cerebral autosomal dominant arteriopathy with subcortical infarcts and leukoencephalopathy; Pcu/CU: precuneus and cuneus

**Fig. 3:**
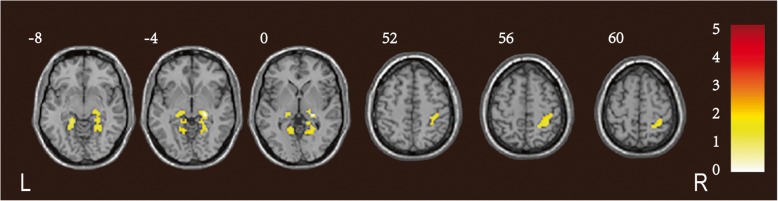
Reductions in the FC between the right Pcu/CU and other brain areas in patients with CADASIL. The CADASIL group exhibited weaker FC between the right Pcu/CU and both PHGs, and between the right Pcu/CU and right postcentral gyrus, compared to controls. The *t-*values are color-coded. FC: functional connectivity; Pcu/CU: precuneus and cuneus; CADASIL: cerebral autosomal dominant arteriopathy with subcortical infarcts and leukoencephalopathy; PHG: parahippocampal gyrus

Compared to the controls, patients with CADASIL exhibited significantly lower ReHos in the right precuneus and cuneus (Pcu/CU), visual association cortex, calcarine gyri, posterior cingulate and limbic lobe (Fig. 2; Table 5). We compared the FC of the right Pcu/CU to those of other brain regions in the CADASIL and control groups. Compared to the controls, the former group exhibited weaker FC between the right Pcu/CU and the bilateral parahippocampal gyrus (PHG), and between the right Pcu/CU and the right postcentral gyrus (Fig. 3; Table 5).

**Table 5:** Significant inter-group differences in ReHo and seed-based FC analysis between CADASIL patients and controls

Predominant cluster regions	Cluster size	Peak *T* value	MNI coordinates			Cluster level *P*FWE-corr
			x	y	z	
ReHo reduction in CADASIL patients						
Right Pcu/CU	155	−5.33	6	−78	30	0.009
Right visual association cortex						
Right calcarine gyri						
Right posterior cingulate						
Right limbic lobe						
FC reductions in CADASIL patients (seed region: right Pcu/CU)						
Right PHG	206	−5.62	24	−27	−3	0.005
Left PHG	171	−4.59	−18	−39	−12	0.011
Right postcentral gyrus	115	−4.14	33	−39	57	0.044

The ReHo clusters and seed-based FC that survived were assigned thresholds of *p* < 0.001 and FWE-corrected to *p* < 0.05 at the cluster level

*ReHo* Regional homogeneity, *FC* Functional connectivity, *CADASIL* Cerebral autosomal dominant arteriopathy with subcortical infarcts and leukoencephalopathy, *MNI* Montreal Neurological Institute, *FWE* Family wise errors, *Pcu/CU* Precuneus and cuneus, *PHG* Parahippocampa gyrus

### Correlations with clinical scores

**Fig. 4:**
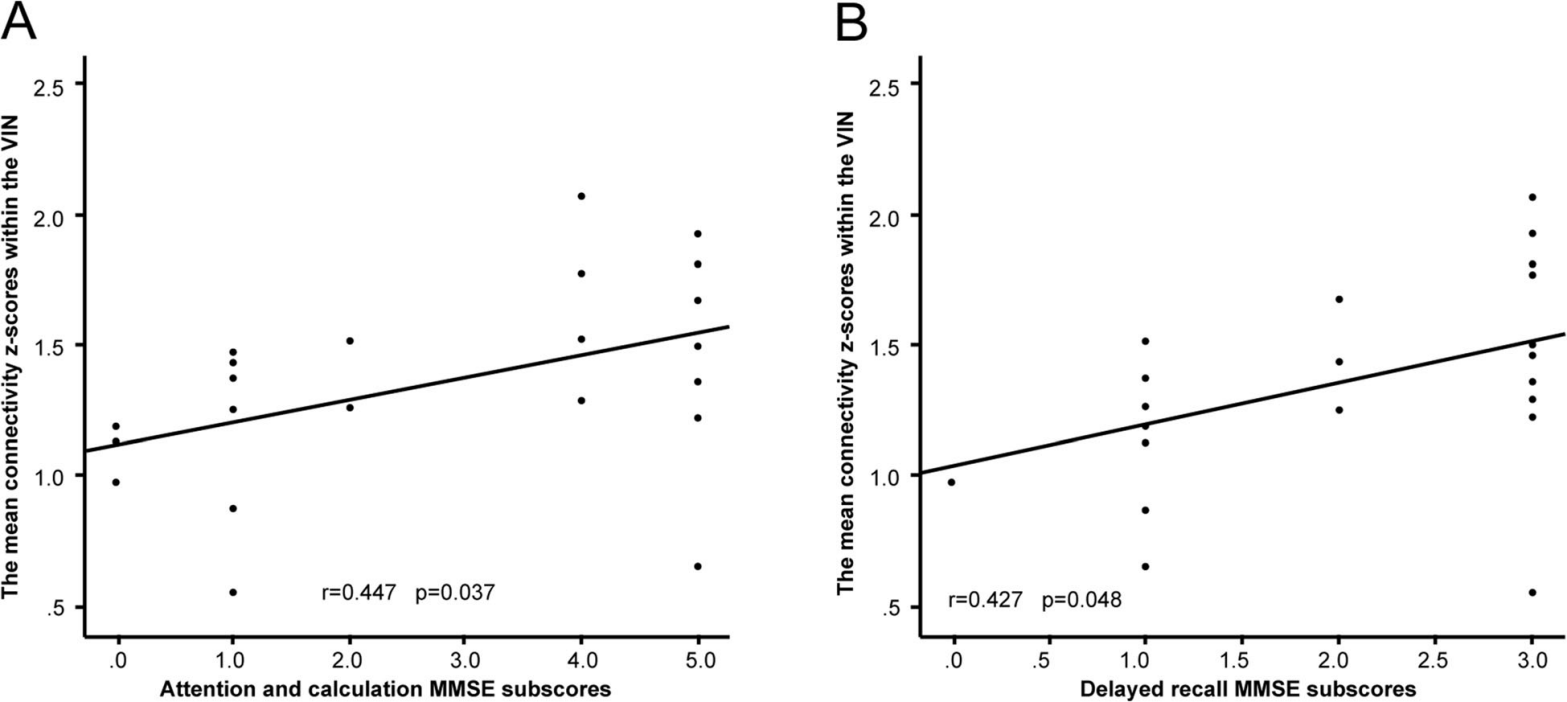
Significant correlations between the mean connectivity z-scores of the LG and CU within the VIN and cognitive measures for surviving clusters in the CADASIL group. **a** The mean connectivity z-scores within the VIN and the attention and calculation MMSE subscores; *r* = 0.447, *p* = 0.037. **b** The mean connectivity z-scores within the VIN and the delayed recall MMSE subscores; *r* = 0.427, *p* = 0.048. LG: lingual gyrus; CU: cuneus; VIN: visual network; CADASIL: cerebral autosomal dominant arteriopathy with subcortical infarcts and leukoencephalopathy; MMSE: Mini-Mental State Examination

The mean connectivity z-scores of the bilateral LG and the right CU within the VIN were positively correlated with both of the attention and calculation (*r* = 0.447, *p* = 0.037), and delayed recall (*r* = 0.427, *p* = 0.048) scores, as revealed by the cognitive domains explored by the MMSE (Fig. 4). There was no correlation between the mean ReHo z-scores of the right Pcu/CU and clinical scores, or between the mean connectivity z-scores within the functional networks and any of the scores on the neurological deficits, depression, or anxiety scales.

## Discussion

ICA revealed reduced intra-network connectivity in the VIN and FRN of CADASIL patients, marked decreases in the ReHos of the right Pcu/CU, visual association cortex, calcarine gyri, posterior cingulate, limbic lobe, and weaker FC between the right Pcu/CU and other brain areas. Furthermore, the mean connectivity z-scores of the dysfunctional areas within the VIN were positively associated with the several cognitive domains of MMSE.

Human executive functions are not mediated by a single brain region, but rather, reflect the dynamic interplay of multiple networks [28–32]. A hallmark of executive function is the ability of rapidly arbitrary links between visual inputs, on the one hand, and actions and goals, on the other, by the use of the learned informations (e.g., applying the brakes of a car when a person is seen ahead) [29]. This ability is termed arbitrary visuomotor mapping; large-scale brain networks engage in reconfiguration and dynamic integration and occipital–parietal–frontal cortical and cortico-subcortical FC networks are involved. First, the visual and parietal regions coordinate with the sensorimotor and premotor areas. Second, the dorsal frontoparietal circuit links to the sensorimotor and frontostriatal networks. Finally, the cortico-cortical interhemisphere coordinates the bilateral sensorimotor regions [20]. During this process, the posterior parietal area, particularly the Pcu, plays a key role in arranging visuomotor planning and using of visual information in movement. The premotor areas serve as relays from the posterior parietal areas to the medial prefrontal cortices, which play receptor roles [33]. The networks exhibit temporal evolution, commencing with the processing of visual information, which is followed by emergence of a visuomotor plan and then action [34]. We found that the intra-network connectivity of the VIN and FRN were reduced in the patient group; the VIN and FRN lie in the occipital and frontoparietal areas, respectively, and include visual regions such as the LG and CU, motor areas such as the IFG and precentral gyrus, and the posterior parietal Pcu. We suggest that all visual input, motor output, and visuomovement transformation may be interrupted in CADASIL patients, thereby affecting arbitrary visuomotor mapping (a form of acquired instrumental behavior) in such patients.

Cortical network nodes are involved in arbitrary visuomotor mapping. The Pcu and CU lie on both sides of the parieto-occipital fissure, in the posterior parietal and inferior occipital lobes, respectively. Resting-state fMRI has revealed three distinct FC patterns in the Pcu. The anterior Pcu is functionally connected with the postcentral and precentral gyri, the sensorimotor regions. The central Pcu connects with the dorsolateral and dorsomedial prefrontal cortex (a cognitive/associative region) and the posterior Pcu with the adjacent, visual cortical regions [35]. The FC data showed that the Pcu serves as a bridge connecting visual perceptions with action during the visuomotor process, being involved in both visuomotor planning and transmission of visual information from the occipital lobe to sensorimotor regions [36–40]. Furthermore, the CU exhibited significant, contralateral visual selectivity when engaged in processing of visual information [35]. Therefore, the Pcu/CU are considered (respectively) the node and hub of the frontoparietal, central executive network involved in arbitrary visuomotor mapping [41]. In the current study, we found that the ReHos of the right Pcu/CU were decreased in CADASIL patients, suggesting that Pcu/CU-mediated executive cognition might be impaired. The functional changes in the Pcu/CU were similar to those of patients with sporadic, subcortical vascular dementia (SVD) [41, 42], indicating that Pcu/CU dysfunction might explain the executive impairments of both hereditary and sporadic SVD.

We found that the intra-network connectivity of the right IFG (within the FRN) was also reduced in CADASIL patients. The IFG plays a vital role in implementing the strategies of multi-component behaviors [43]. Magnetoencephalography revealed that the IFG connected directly with the visual and motor cortices when a visuomotor, precision, grip force task was underway. This network may effectively complement the traditional step-by-step processing of the occipital-parietal-premotor-motor pathway during such tasks [44]. We found that two pathways connecting the visual and motor cortices during visuomotor behavior might be affected in CADASIL patients; the pathways involve the traditional frontoparietal nodes (the Pcu/CU) and the IFG.

We also found that patients exhibited weaker FC between the right Pcu/CU and the bilateral PHG, and between the right Pcu/CU and the right postcentral gyrus (the primary somatosensory area), than controls. Matyas et al. used the mouse whisker model to show that cortical motor control was driven by the primary somatosensory cortex, which also delivered rapid negative feedback during sensorimotor integration [45]. Similar to the expansion of motor control into the sensory cortex, the PHG lies in the medial-temporal lobe and processes visual motion such as speed, acceleration, and the direction of hand movement, but only during a visuomotor task. Thus, the PHG may engage in visuomotor integration [46, 47]. In general, the poor FC between the Pcu/CU, on the one hand, and the postcentral gyrus and the PHG, on the other, suggest that both coordination and integration of arbitrary sensorimotor associations are dysfunctional in CADASIL patients.

We found positive associations between the mean connectivity z-scores of the LG and CU within the VIN, and the scores on different cognitive domains of the MMSE including those reflecting attention, calculation and delayed recall. Patients with the most serious cognitive impairments may exhibit lower VIN connectivity. In a sense, such decreases render it easier to detect severe cognitive issues in patients with CADASIL.

Our work had certain limitations. First, we only studied a small sample of CADASIL patients. Second, we only used the MMSE and MoCA to evaluate cognitive function. Future studies with larger sample sizes should focus on more detailed cognitive domains such as executive function and processing speed.

ALFF and ReHos are two quantitative methods used to evaluate local spontaneous neuronal activity. ALFF reflects the intensity of neuronal activity within a single voxel, and ReHos indicate the temporal similarity of neuronal activity between a single voxel and its neighboring voxels [18, 21]. In our recent resting-state ALFF analyses, we found that CADASIL exhibited lower ALFF values in the right Pcu/CU [18]. In contrast, the present study revealed lower ReHos in the right Pcu/CU and other areas including visual association cortex, calcarine gyri, posterior cingulate, limbic lobe. More importantly, we also found that CADASIL exhibited decreased intra-network connectivity within the VIN and FRN. These results suggest that CADASIL may exhibit dysfunctional visuomotor behaviors, a hallmark of executive function.

## Conclusions

In this study, we found aberrant resting-state brain functional network connectivity within the VIN and FRN, and lower ReHos, in patients with CADASIL. These may explain the arbitrary visuomotor behaviors of such patients, reflecting (downward) transitions in visual stimuli, movement location, and execution response mapping. The work advances our understanding of the executive cognition architectures underlying CADASIL, and will aid in the development of therapeutic strategies.

## Data Availability

The datasets used and/or analysed during the current study are available from the corresponding author on reasonable request.

## Abbreviations

CADASIL: Cerebral autosomal dominant arteriopathy with subcortical infarcts and leukoencephalopathy
FC: Functional connectivity
fMRI: Functional magnetic resonance imaging
FRN: Frontal network
MMSE: Mini-Mental State Examination
Pcu/CU: Precuneus and cuneus
ReHo: Regional homogeneity
VIN: Visual network

## References

[1] Chabriat H, Joutel A, Dichgans M (2009). Cadasil. Lancet Neurol.

[2] Buffon F, Porcher R, Hernandez K (2006). Cognitive profile in CADASIL. J Neurol Neurosurg Psychiatry.

[3] Chabriat H, Vahedi K, Iba-Zizen MT (1995). Clinical spectrum of CADASIL: a study of 7 families. Cerebral autosomal dominant arteriopathy with subcortical infarcts and leukoencephalopathy. Lancet.

[4] Jouvent E, Duchesnay E, Hadj-Selem F (2016). Prediction of 3-year clinical course in CADASIL. Neurology.

[5] Gunda B, Hervé D, Godin O (2012). Effects of gender on the phenotype of CADASIL. Stroke.

[6] Chabriat H, Hervé D, Duering M (2016). Predictors of clinical worsening in cerebral autosomal dominant arteriopathy with subcortical infarcts and leukoencephalopathy: prospective cohort study. Stroke.

[7] Ling Y, De Guio F, Duering M (2017). Predictors and clinical impact of incident lacunes in cerebral autosomal dominant arteriopathy with subcortical infarcts and leukoencephalopathy. Stroke.

[8] Liem MK, van der Grond J, Haan J (2007). Lacunar infarcts are the main correlate with cognitive dysfunction in CADASIL. Stroke.

[9] Viswanathan A, Gschwendtner A, Guichard JP (2007). Lacunar lesions are independently associated with disability and cognitive impairment in CADASIL. Neurology.

[10] Peters N, Holtmannspötter M, Opherk C (2006). Brain volume changes in CADASIL: a serial MRI study in pure subcortical ischemic vascular disease. Neurology.

[11] Shi Y, Li S, Li W (2018). MRI lesion load of cerebral small vessel disease and cognitive impairment in patients with CADASIL. Front Neurol.

[12] Jouvent E, Reyes S, De Guio F (2015). Reaction time is a marker of early cognitive and behavioral alterations in pure cerebral small vessel disease. J Alzheimers Dis.

[13] Delorme S, De Guio F, Reyes S (2017). Reaction time is negatively associated with Corpus callosum area in the early stages of CADASIL. AJNR Am J Neuroradiol.

[14] O'Sullivan M, Barrick TR, Morris RG (2005). Damage within a network of white matter regions underlies executive dysfunction in CADASIL. Neurology.

[15] O'Sullivan M, Singhal S, Charlton R (2004). Diffusion tensor imaging of thalamus correlates with cognition in CADASIL without dementia. Neurology.

[16] Craggs LJ, Yamamoto Y, Ihara M (2014). White matter pathology and disconnection in the frontal lobe in cerebral autosomal dominant arteriopathy with subcortical infarcts and leukoencephalopathy (CADASIL). Neuropathol Appl Neurobiol.

[17] Cullen B, Moreton FC, Stringer MS (2016). Resting state connectivity and cognitive performance in adults with cerebral autosomal-dominant arteriopathy with subcortical infarcts and leukoencephalopathy. J Cereb Blood Flow Metab.

[18] Su J, Wang M, Ban S (2019). Relationship between changes in resting-state spontaneous brain activity and cognitive impairment in patients with CADASIL. J Headache Pain.

[19] Gavazzi Gioele, Orsolini Stefano, Salvadori Emilia, Bianchi Andrea, Rossi Arianna, Donnini Ida, Rinnoci Valentina, Pescini Francesca, Diciotti Stefano, Viggiano Maria Pia, Mascalchi Mario, Pantoni Leonardo (2019). Functional Magnetic Resonance Imaging of Inhibitory Control Reveals Decreased Blood Oxygen Level Dependent Effect in Cerebral Autosomal Dominant Arteriopathy With Subcortical Infarcts and Leukoencephalopathy. Stroke.

[20] Brovelli A, Badier JM, Bonini F (2017). Dynamic reconfiguration of visuomotor-related functional connectivity networks. J Neurosci.

[21] Zang Y, Jiang T, Lu Y (2004). Regional homogeneity approach to fMRI data analysis. Neuroimage.

[22] Bramell-Risberg E, Jarnlo GB, Elmståhl S (2012). Separate physical tests of lower extremities and postural control are associated with cognitive impairment. Results from the general population study good aging in Skåne (GÅS-SNAC). Clin Interv Aging.

[23] Marengoni A, Bandinelli S, Maietti E (2017). Combining gait speed and recall memory to predict survival in late life: population-based study. J Am Geriatr Soc.

[24] Duering M, Righart R, Csanadi E (2012). Incident subcortical infarcts induce focal thinning in connected cortical regions. Neurology.

[25] Fazekas F, Chawluk JB, Alavi A (1987). MR signal abnormalities at 1.5 T in Alzheimer’s dementia and normal aging. AJR Am J Roentgenol.

[26] Yan CG, Wang XD, Zuo XN (2016). DPABI: data processing & analysis for (resting-state) brain imaging. Neuroinformatics.

[27] Allen EA, Erhardt EB, Damaraju E (2011). A baseline for the multivariate comparison of resting-state networks. Front Syst Neurosci.

[28] Braun U, Schäfer A, Walter H (2015). Dynamic reconfiguration of frontal brain networks during executive cognition in humans. Proc Natl Acad Sci U S A.

[29] Eliassen JC, Souza T, Sanes JN (2003). Experience-dependent activation patterns in human brain during visual-motor associative learning. J Neurosci.

[30] Chouinard PA, Goodale MA (2009). FMRI adaptation during performance of learned arbitrary visuomotor conditional associations. Neuroimage.

[31] Madhavan R, Bansal AK, Madsen JR et al (2018) Neural interactions underlying Visuomotor associations in the human brain. Cereb Cortex. 10.1093/cercor/bhy333 [Epub ahead of print] 10.1093/cercor/bhy333 PMC6917519 30590542

[32] Ester EF, Sprague TC, Serences JT (2015). Parietal and frontal cortex encode stimulus-specific mnemonic representations during visual working memory. Neuron.

[33] Brovelli A, Chicharro D, Badier JM (2015). Characterization of cortical networks and corticocortical functional connectivity mediating arbitrary Visuomotor mapping. J Neurosci.

[34] Cappadocia DC, Monaco S, Chen Y (2017). Temporal evolution of target representation, movement direction planning, and reach execution in occipital-parietal-frontal cortex: an fMRI study. Cereb Cortex.

[35] Margulies DS, Vincent JL, Kelly C (2009). Precuneus shares intrinsic functional architecture in humans and monkeys. Proc Natl Acad Sci U S A.

[36] Grol MJ, de Lange FP, Verstraten FA (2006). Cerebral changes during performance of overlearned arbitrary visuomotor associations. J Neurosci.

[37] Fernandez-Ruiz J, Goltz HC, DeSouza JF (2007). Human parietal “reach region” primarily encodes intrinsic visual direction, not extrinsic movement direction, in a visual motor dissociation task. Cereb Cortex.

[38] Culham JC, Valyear KF (2006). Human parietal cortex in action. Curr Opin Neurobiol.

[39] Grol MJ, Toni I, Lock M (2009). Spatial representation of overlearned arbitrary visuomotor associations. Exp Brain Res.

[40] Gertz H, Fiehler K (2015). Human posterior parietal cortex encodes the movement goal in a pro−/anti-reach task. J Neurophysiol.

[41] Menon V (2011). Large-scale brain networks and psychopathology: a unifying triple network model. Trends Cogn Sci.

[42] Li C, Liu C, Yin X (2014). Frequency-dependent changes in the amplitude of low-frequency fluctuations in subcortical ischemic vascular disease (SIVD): a resting-state fMRI study. Behav Brain Res.

[43] Dippel G, Beste C (2015). A causal role of the right inferior frontal cortex in implementing strategies for multi-component behaviour. Nat Commun.

[44] Papadelis C, Arfeller C, Erla S (2016). Inferior frontal gyrus links visual and motor cortices during a visuomotor precision grip force task. Brain Res.

[45] Matyas F, Sreenivasan V, Marbach F (2010). Motor control by sensory cortex. Science.

[46] Tankus A, Fried I (2012). Visuomotor coordination and motor representation by human temporal lobe neurons. J Cogn Neurosci.

[47] Sato N, Nakamura K (2003). Visual response properties of neurons in the parahippocampal cortex of monkeys. J Neurophysiol.

